# Extending the models for iron and sulfur oxidation in the extreme Acidophile *Acidithiobacillus ferrooxidans*

**DOI:** 10.1186/1471-2164-10-394

**Published:** 2009-08-24

**Authors:** Raquel Quatrini, Corinne Appia-Ayme, Yann Denis, Eugenia Jedlicki, David S Holmes, Violaine Bonnefoy

**Affiliations:** 1 Center for Bioinformatics and Genome Biology, MIFAB, Fundación Ciencia para la Vida and Depto. de Ciencias Biologicas, Facultad de Ciencias de la Salud, Universidad Andres Bello, Santiago, Chile; 2 Laboratoire de Chimie Bactérienne, Institut de Microbiologie de la Méditerranée, Centre National de la Recherche Scientifique, Marseille, France; 3 Platforme Transcriptome, Institut de Microbiologie de la Méditerranée, Centre National de la Recherche Scientifique, Marseille, France; 4 Cellular and Molecular Biology, Faculty of Medicine, University of Chile, Santiago, Chile; 5 Institute for Food Research, Norwich Research Park, Colney Lane, Norwich, NR4 7AU, UK

## Abstract

**Background:**

*Acidithiobacillus ferrooxidans *gains energy from the oxidation of ferrous iron and various reduced inorganic sulfur compounds at very acidic pH. Although an initial model for the electron pathways involved in iron oxidation has been developed, much less is known about the sulfur oxidation in this microorganism. In addition, what has been reported for both iron and sulfur oxidation has been derived from different *A. ferrooxidans *strains, some of which have not been phylogenetically characterized and some have been shown to be mixed cultures. It is necessary to provide models of iron and sulfur oxidation pathways within one strain of *A. ferrooxidans *in order to comprehend the full metabolic potential of the pangenome of the genus.

**Results:**

Bioinformatic-based metabolic reconstruction supported by microarray transcript profiling and quantitative RT-PCR analysis predicts the involvement of a number of novel genes involved in iron and sulfur oxidation in *A. ferrooxidans *ATCC23270. These include for iron oxidation: *cup *(copper oxidase-like), *ctaABT *(heme biogenesis and insertion), *nuoI *and *nuoK *(NADH complex subunits), *sdrA1 *(a NADH complex accessory protein) and *atpB *and *atpE *(ATP synthetase F0 subunits). The following new genes are predicted to be involved in reduced inorganic sulfur compounds oxidation: a gene cluster (*rhd, tusA, dsrE, hdrC, hdrB, hdrA, orf2, hdrC, hdrB*) encoding three sulfurtransferases and a heterodisulfide reductase complex, *sat *potentially encoding an ATP sulfurylase and *sdrA2 *(an accessory NADH complex subunit). Two different regulatory components are predicted to be involved in the regulation of alternate electron transfer pathways: 1) a gene cluster (*ctaRUS*) that contains a predicted iron responsive regulator of the Rrf2 family that is hypothesized to regulate cytochrome *aa*_3 _oxidase biogenesis and 2) a two component sensor-regulator of the RegB-RegA family that may respond to the redox state of the quinone pool.

**Conclusion:**

Bioinformatic analysis coupled with gene transcript profiling extends our understanding of the iron and reduced inorganic sulfur compounds oxidation pathways in *A. ferrooxidans *and suggests mechanisms for their regulation. The models provide unified and coherent descriptions of these processes within the type strain, eliminating previous ambiguity caused by models built from analyses of multiple and divergent strains of this microorganism.

## Background

*Acidithiobacillus ferrooxidans *is an acidophilic, chemolithoautotrophic γ-proteobacterium that uses energy and electrons derived from the oxidation of ferrous iron (Fe(II)) and reduced inorganic sulfur compounds (RISCs) for carbon dioxide and nitrogen fixation and other anabolic processes. It is a member of a consortium of microorganisms used for industrial copper recovery (bioleaching or biomining) and gold recovery (biooxidation) and contributes to the geobiochemical recycling of metals and nutrients in pristine and contaminated acid-rich environments.

When *A. ferrooxidans *requires reducing power it is confronted by a particularly challenging bioenergetic problem because the standard reduction half-potential of the Fe(II)/Fe(III) couple (+0.77 V at pH 2, the pH of the medium) is much more positive than that of the NAD(P)/NAD(P)H couple (-0.32 V at the cytoplasmic pH 7). This means that electrons have to be "pushed uphill" from Fe(II) to NAD(P) against the redox potential gradient (uphill pathway). The energy to accomplish this was proposed to come from the proton motive force that naturally occurs across the periplasmic membrane of *A. ferrooxidans *(outside = pH 2, inside = pH 7). It was further suggested that protons, entering the cell through a membrane associated ATP synthetase, could be consumed during the reduction of oxygen to water (2e^- ^+ 1/2 O_2 _+ 2H^+ ^→ H_2_O) in which electrons for this reaction come from the oxidation of Fe(II) in a pathway that is thermodynamically favorable (downhill pathway). These ideas were first promulgated based upon theoretical considerations by Ingledew [1] but the model has been extended more recently and major parts of it are now supported by experimental evidence (reviewed in [2]).

**Figure 1:**
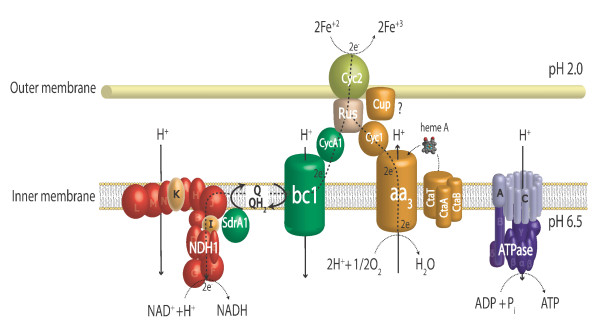
**Model of Fe(II) oxidation in *A. ferrooxidans *ATCC 23270**. The flow of electrons is shown from the oxidation of Fe^+2 ^by Cyc2 to reduce oxygen via the *aa*_3 _complex (downhill pathway) or to reduce NAD+ via *bc*_1_/quinone pool/NADH complex (uphill pathway). The downhill pathway can consume protons entering via the ATPase complex helping to drive ATP synthesis or via the *bc*_1_/quinone pool/NADH complex that drives the flow of electrons in the uphill pathway. The switch point between the downhill and uphill flow is suggested to be at the level of rusticyanin (Rus). Abbreviations used can be found in the text.

According to the model (see Figure 1), components involved in the downhill electron pathway from Fe(II) to oxygen include: an outer membrane high molecular-weight cytochrome *c *encoded by *cyc2*, a gene of unknown function (ORF1), a periplasmic soluble blue copper protein rusticyanin encoded by *rus*, a periplasmic membrane-bound dihemic cytochrome *c*_4 _encoded by *cyc1 *and a terminal cytochrome oxidase of the *aa*_3_-type encoded by the *coxBACD *gene cluster [3-6]. In *A. ferrooxidans *ATCC 33020 these genes are cotranscribed in an eight gene transcriptional unit, termed the *rus *operon that is upregulated in Fe(II)-grown cells [5,7]. Gene context analysis of the recently annotated genomic sequence of *A. ferrooxidans *type strain ATCC 23270 demonstrates that the *rus *operon is similarly organized in this strain [8].

The uphill components include: a cytochrome *bc*_1 _complex (complex III, ubiquinol-cytochrome *c *reductase), the quinone pool and a NADH1 dehydrogenase complex (hereafter abbreviated to NADH complex) functioning in reverse [9-11]. Genetic and biophysical evidence obtained for *A. ferrooxidans *ATCC 19859 and ATCC 33020 support this view [11-13]. In these two strains, the genes encoding the *bc*_1 _complex have been shown to be part of a five-gene operon, termed the *petI *operon, mainly transcribed in Fe(II) conditions and organized as following: a diheme cytochrome *c*_4 _(*cycA1*), a short-chain dehydrogenase (*sdrA1*) of unknown function, a cytochrome *b *(*petA1*), an iron-sulfur protein Rieske (*petB1*), and a cytochrome *c*_1 _(*petC1*) [2,12,13]. Based on the cotranscription of the diheme cytochrome *c*_4 _gene (*cycA1*) with those encoding the *bc*_1 _complex, it was proposed that electrons from the diheme cytochrome *c*_4 _take a thermodynamically uphill pathway via the *bc*_1 _complex to a NADH complex driven energetically by proton motive force [2,12-14] (see Figure 1). Adjacent to the *petI *operon, is a three gene (*resBC *and a hypothetical gene) operon in which ResBC are predicted to be chaperones involved in heme insertion in *c*-type cytochromes, possibly those involved in the maturation of the *c*_1 _cytochrome of the *bc*_1 _complex [12]. The *petI *operon is similarly organized and transcribed in *A. ferrooxidans *ATCC 23270 [14] supporting this model.

The bifurcation in the flow of electrons from Fe(II) to reduce either NAD (uphill) or O_2 _(downhill) has been proposed to occur at the level of rusticyanin, a small periplasmic blue copper protein [2,13,14] (see Figure 1). By adjusting the flow of electrons at this branch point, it was suggested that *A. ferrooxidans *could balance its requirements for NADH and ATP [9].

The components involved in both the uphill and downhill electron flow have been shown to form a Fe-oxidizing/O_2_-reducing supercomplex spanning both inner and outer membranes in an unnamed strain of *A. ferrooxidans *[15] supporting previous models. The supercomplex has also been suggested to include a copper containing protein (ORF1) physically associated with the periplasmic Cyc1 that is proposed to be involved in downhill electron flow. In addition, *in vitro *reconstitution of the iron oxidation system of the *A. ferrooxidans *type strain with rusticyanin, *aa*_3_-type cytochrome oxidase, the cytochromes *c *Cyc2 and Cyc1 has been reported recently [16].

Despite progress in understanding iron oxidation in the *Acidithiobacillus *genus, several lacunae in our knowledge persist, such as the identification of components involved in the proposed connection between the *bc*_1_/quinone complex and the NADH complex, the identification of the chaperones used for cofactor insertion into the relevant redox proteins, and the mechanisms and components regulating the electron flux via the downhill pathway to O_2 _versus the uphill pathway to the NADH complex.

Reduced inorganic sulfur oxidation is widespread in prokaryotes (reviewed in [17,18]. However, in contrast to iron that occurs in only two oxidation states, sulfur exists in multiple states from -2 to +6, complicating identification of intermediates and relevant enzymes in sulfur oxidation. Also, some sulfur compounds can be oxidized abiotically adding further difficulties in resolving enzymatic steps from chemical changes. Despite these difficulties, several biological pathways for RISCs oxidation have been identified including the phylogenetically widespread sulfur oxidizing (*sox*) gene pathway (reviewed in [17]) and the archaeal type sulfur oxygenase reductase (*sor*) gene system (reviewed in [19]). However, neither *sox*, nor *sor *have been detected in the genome of *A. ferrooxidans *[2,8], raising the question as to how this organism oxidizes RISCs.

Much of our knowledge of RISCs oxidation in *A. ferrooxidans *comes from enzyme assays performed many years ago on different strains, some of which await phylogenetic characterization or, in some cases, have not yet even been isolated as pure cultures [2]. Those that have been characterized form genetically diverse clusters with at least three phylogenetic groups [20-23]. This raises the possibility that current models of RISCs oxidation reflect a patchwork assemblage of predicted pathways and activities that may not exist *in toto *in any one *A. ferrooxidans *strains and perhaps more accurately reflect the pangenomic capacity of the genus *Acidithiobacillus *for RISCs oxidation.

Thus the current investigation was prompted by the need to generate a more comprehensive picture of iron and sulfur bioenergetics by searching for missing steps and predicting novel enzymatic and electron transfer components and to provide a coherent picture of these processes in one strain of *A. ferrooxidans *(type strain, ATCC 23270), facilitating the recognition of species variation in bioenergetic pathways.

## Results and Discussion

### General Features of the Transcriptional Profiles

RNA, isolated from mid-log *A. ferrooxidans *grown in either sulfur (S^0^) or ferrous iron (Fe(II)) medium, was used to probe gene expression using microarrays displaying unique oligonucleotides representing about 3000 predicted genes of the *A. ferrooxidans *type strain genome. Using statistical criteria described previously [14], a 1.5 log ratio of median cut-off (corresponding to genes induced more than 2.8 fold) was selected as indicating differential gene expression in the two growth conditions (Table 1[7,24,25], Table 2[24-29] and Additional file 1[7,24-26,29]). The expression patterns observed with the microarrays were validated for some relevant genes by real-time quantitative PCR (Table 3[7,24-26,28,29]). One hundred and ninety four genes presented a differential expression profile, of which 110 were upregulated (up to 38 fold) while 84 were downregulated (up to 10 fold) in iron compared to sulfur medium. Genes exhibiting differential expression were grouped by hierarchical clustering and were found to be mostly associated with unknown functions, energy metabolism, cell envelope and central intermediary processes (Additional file 1[7,24-26,29]).

**Table 1:** Microarray expression data for iron induced genes

ID NC011761	Gene	Function	log_2 _ratio median	One sample t-Test (p-value)	Proteomic data: strain/references
***pet I *operon**					
AFE_3111	*petC1*	ubiquinol-cytochrome *c *reductase, cytochrome *c*1 subunit	3,7	0,00*	
AFE_3110	*petB1*	ubiquinol-cytochrome *c *reductase, cytochrome *b *subunit	4,4	0,00*	
AFE_3109	*petA1*	ubiquinol-cytochrome *c *reductase, iron-sulfur subunit	3,7	0,00*	CCM 4252/[24]
AFE_3108	*sdrA1*	oxidoreductase, short-chain dehydrogenase/reductase family	3,9	0,00*	
AFE_3107	*cycA1*	cytochrome *c*4	3,7	0,00*	
***rus *operon**					
AFE_3153	*cyc2*	cytochrome *c*	2,5	0,00*	ATCC 33020/[7]

AFE_3152	*cyc1*	cytochrome *c*552	2,8	0,00*	ATCC 33020/[7] CCM 4253/[24] ATCC 19859/[25]

AFE_3151	*cup*	conserved hypothetical protein	3,1	0,00*	ATCC 33020/[7]
AFE_3150	*coxB*	cytochrome *c *oxidase, *aa*3-type, subunit II	2,9	0,00*	ATCC 33020/[7]
AFE_3149	*coxA*	cytochrome *c *oxidase, *aa*3-type, subunit I	2,5	0,00*	ATCC 33020/[7]
AFE_3148	*coxC*	cytochrome *c *oxidase, *aa*3-type, subunit III	1,7	0,02*	ATCC 33020/[7]

AFE_3146	*rus*	rusticyanin	1,6	0,00*	ATCC 33020/[7] CCM 4253/[24] ATCC 19859/[25]

AFE_3146	*rus*	rusticyanin	2,1	0,00*	ATCC 33020/[7] CCM 4253/[24] ATCC 19859/[25]

AFE_3146	*rus*	rusticyanin	1,9	0,00*	ATCC 33020/[7] CCM 4253/[24] ATCC 19859/[25]

**Cytochrome *c *oxidase complex biogenesis operon**					
AFE_3144	*ctaA*	heme A synthase	2,4	0,00*	
AFE_3143	*ctaB*	heme O synthase, protoheme IX farnesyltransferase	0,4	0,18	
AFE_3142	*ctaT*	major facilitator family transporter	0,8	0,00*	
AFE_3141	*ctaR*	iron responsive regulator of the Rrf2 family	ND	ND	
AFE_3139	*ctaU*	hypothetical protein	1,8	0,00*	
AFE_3138	*ctaS*	oxidoreductase, 2OG-Fe(II) oxygenase family	1,4	0,00*	
**Sensor/regulator two-component signal transduction system**					
AFE_3137	*regA*	DNA-binding response regulator	0,7	0,02	
AFE_3136	*regB*	sensor histidine kinase	2,0	0,00*	
**NADH complex operon**					
AFE_2630	*nuoA*	NADH-quinone oxidoreductase, A subunit	-0,1	0,80	
AFE_2629	*nuoB*	NADH-quinone oxidoreductase, B subunit	0,3	0,13	
AFE_2628	*nuoC*	NADH-quinone oxidoreductase, C subunit	0,0	0,69	
AFE_2627	*nuoD*	NADH-quinone oxidoreductase, D subunit	-0,5	0,64	
AFE_2626	*nuoE*	NADH-quinone oxidoreductase, E subunit	-1,1	0,05	
AFE_2625	*nuoF*	NADH-quinone oxidoreductase, F subunit	0,4	0,01	
AFE_2624	*nuoG*	NADH-quinone oxidoreductase, G subunit	-0,2	0,24	
AFE_2623	*nuoH*	NADH-quinone oxidoreductase, H subunit	-0,7	0,00	
AFE_2622	*nuoI*	NADH-quinone oxidoreductase, I subunit	1,4	0,00*	
AFE_2621	*nuoJ*	NADH-quinone oxidoreductase, J subunit	-0,1	0,21	
AFE_2620	*nuoK*	NADH-quinone oxidoreductase, K subunit	0,9	0,03*	
AFE_2619	*nuoL*	NADH-quinone oxidoreductase, L subunit	-0,9	0,00	
AFE_2618	*nuoM*	NADH-quinone oxidoreductase, M subunit	-0,8	0,00	
AFE_2617	*nuoN*	NADH-quinone oxidoreductase, N subunit	-0,4	0,19	
**ATP synthetase complex operon**					
AFE_3209	*atpB*	ATP synthase F0, A subunit	1,8	0,00*	
AFE_3208	*atpE*	ATP synthase F0, C subunit	1,4	0,00*	
AFE_3207	*atpF*	ATP synthase F0, B subunit	0,4	0,01	
AFE_3206	*atpH*	ATP synthase F1, delta subunit	0,4	0,02	
AFE_3205	*atpA*	ATP synthase F1, alpha subunit	-0,2	0,99	
AFE_3204	*atpG*	ATP synthase F1, gamma subunit	-0,5	0,23	
AFE_3203	*atpD*	ATP synthase F1, beta subunit	0,0	0,75	
AFE_3202	*atpC*	ATP synthase F1, epsilon subunit	-0,6	0,01	

Gene expression values (log_2 _ratio of median) for all genes/operons alluded in the revised model of Fe(II) oxidation in *A. ferrooxidans *ATCC 23270. Genes with a log_2 _ratio of median larger than |1.5| (corresponding to genes induced more than 2.8 fold) are considered differentially expressed (indicated with *) and genes p-value <0,005 are considered significant. Gene ID is that of Genbank genome annotation NC 011761. The reference and the strain in which the level of the gene product has been shown to be higher in Fe(II) than in S^0 ^conditions are indicated in the last column. ND: not determined.

**Table 2:** Microarray expression data for sulfur induced genes

ID NC011761	Gene	Function	log_2 _ratio median	One sample t-Test (p-value)	Proteomic data: strain/references
***pet II *operon**					

AFE_2732	*hip*	High potential iron-sulfur protein	-1,8	0,00*	

AFE_2731	*petC2*	ubiquinol-cytochrome *c *reductase, cytochrome *c*1 subunit	-0,3	0,09	

AFE_2730	*petB2*	ubiquinol-cytochrome *c *reductase, cytochrome *b *subunit	-1,7	0,00*	

AFE_2729	*petA2*	ubiquinol-cytochrome *c *reductase, iron-sulfur subunit	-1,9	0,00*	

AFE_2728	*sdrA2*	oxidoreductase, short-chain dehydrogenase/reductase family	-1,1	0,00*	

AFE_2727	*cycA2*	cytochrome *c*4	-0,5	0,17	

**Heterodisulfide reductase complex operon**					

AFE_2586	*hdrB*	heterodisulfide reductase subunit B, homolog	-1,5	0,00*	

AFE_2558	*rhd*	rhodanese-like domain protein	0,1	0,38	

AFE_2557	*tusA*	conserved hypothetical protein	-2,4	0,00*	

AFE_2556	*dsrE*	conserved hypothetical protein	-2,1	0,00*	

AFE_2555	*hdrC*	iron-sulfur cluster-binding protein	-2,1	0,00*	

AFE_2554	*hdrB*	heterodisulfide reductase subunit B, homolog	-2,0	0,00*	

AFE_2553	*hdrA*	pyridine nucleotide-disulfide oxidoreductase	-2,6	0,00*	

AFE_2552	*orf2*	conserved hypothetical protein	ND	ND	

AFE_2551	*hdrC*	iron-sulfur cluster-binding protein	-2,4	0,00*	

AFE_2550	*hdrB*	succinate dehydrogenase/fumarate reductase, C subunit	-1,9	0,00*	

**Sulfide-quinone reductase**					

AFE_1792	*sqr*	sulfide-quinone reductase, putative	-1,6	0,00*	CCM 4253/[24] NASF-1/[26]

**Cytochrome *bd *ubiquinol oxidase**					

AFE_0955	*cydA*	cytochrome *d *ubiquinol oxidase, subunit I	-2,0	0,00*	

AFE_0954	*cydB*	cytochrome *d *ubiquinol oxidase, subunit II	-2,6	0,00*	

**Cytochrome *bo*3 ubiquinol oxidase**					

AFE_0634	*cyoD*	cytochrome *o *ubiquinol oxidase, subunit IV	-2,3	0,00*	

AFE_0633	*cyoC*	cytochrome *o *ubiquinol oxidase, subunit III	-3,0	0,00*	

AFE_0632	*cyoB*	cytochrome *o *ubiquinol oxidase, subunit I	-2,7	0,00*	

AFE_0631	*cyoA*	cytochrome *o *ubiquinol oxidase, subunit II	-3,2	0,00*	

**Sulfate adenylyltransferase**					

AFE_0539	*sat*	sulfate adenylyltransferase, putative/adenylylsulfate kinase	0,7	0,00*	

**Thiosulfate-quinone oxidoreductase complex operon**					

AFE_0046		conserved hypothetical protein	-1,7	0,00*	

AFE_0045		sulfur/pyrite/thiosulfate/sulfide-induced protein	-1,1	0,00*	ATCC 19859/[25] ATCC 19859/[28]

AFE_0044	*doxDA*	Thiosulfate-quinone oxidoreductase, DoxD-like family protein	-2,3	0,00*	

AFE_0043		periplasmic solute-binding protein, putative	-2,2	0,00*	CCM 4253/[24] ATCC 23270/[25] ATCC 23270/[29]

AFE_0042		Tat pathway signal sequence domain protein	-1,5	0,00*	

AFE_0041		C4-dicarboxylate transporter/malic acid transport protein	-1,5	0,00*	

**Tetrathionate hydrolase**					

AFE_0029	*tetH*	Tetrathionate hydrolase	-0,6	0,22*	ATCC 23270/[27]

Gene expression values (log_2 _ratio of median) for all genes/operons alluded in the revised model of sulfur oxidation in *A. ferrooxidans *ATCC 23270. Genes with a log_2 _ratio of median larger than |1.5| (corresponding to genes induced more than 2.8 fold) are considered differentially expressed (indicated with *) and genes p-value <0,005 are considered significant. Gene ID is that of Genbank genome annotation NC 011761. The reference and the strain in which the level of the gene product has been shown to be higher in S^0 ^than in Fe(II) conditions are indicated in the last column.

**Table 3:** Q-PCR expression data for relevant validated genes

ID NC011761	Gene (locus)	Function	log_2 _(Fe/S)	Induced	Proteomic data: strain/references
***rus *operon**					

AFE_3146	*rus*	rusticyanin	3,8	Fe	ATCC 33020/[7] CCM 4253/[24] ATCC 19859/[25]

AFE_3151	*cup*	conserved hypothetical protein	4,9	Fe	ATCC 33020/[7]

**Cytochrome *c *oxidase complex biogenesis operon**					

AFE_3141	*ctaR*	iron responsive regulator of the Rrf2 family	3,9	Fe	

**Sensor/regulator two-component signal transduction system**					

AFE_3137	*regA*	DNA-binding response regulator	4,3	Fe	

***petI *operon**					

AFE_3108	*sdrA1*	oxidoreductase, short-chain dehydrogenase/reductase family	3,7	Fe	

AFE_3109	*petA1*	ubiquinol-cytochrome *c *reductase, iron-sulfur subunit	6,4	Fe	CCM 4252/[24]

AFE_3110	*petB1*	ubiquinol-cytochrome *c *reductase, cytochrome *b *subunit	5,7	Fe	

AFE_3111	*petC1*	ubiquinol-cytochrome *c *reductase, cytochrome *c*1 subunit	5,5	Fe	

**Others**					

AFE_2599	-		1,2	Fe	

AFE_3116	-		1,2	Fe	

AFE_3119	-		3,4	Fe	

AFE_3124	*cysD*	sulfate adenylyltransferase, small subunit	4,8	Fe	CCM 4253/[24]

**Thiosulfate-quinone oxidoreductase complex operon**					

AFE_0043	-	periplasmic solute-binding protein, putative	-2,5	S	CCM 4253/[24] ATCC 19859/[25] ATCC 23270/[29]

AFE_0045	-	sulfur/pyrite/thiosulfate/sulfide-induced protein	-1,2	S	ATCC 19859/[25] ATCC 19859/[28]

**Cytochrome *bd *ubiquinol oxidase**					

AFE_0955	*cydA*	cytochrome *d *ubiquinol oxidase, subunit I	-2,0	S	

**Cytochrome *bo*3 ubiquinol oxidase**					

AFE_0632	*cyoB*	cytochrome *o *ubiquinol oxidase, subunit I	-3,2	S	

**Heterodisulfide reductase complex operon**					

AFE_2553	*hdrA*	pyridine nucleotide-disulfide oxidoreductase	-1,3	S	

AFE_2555	*hdrC*	iron-sulfur cluster-binding protein	-1,6	S	

AFE_2586	*hdrB*	heterodisulfide reductase subunit B, homolog	-0,8	S	

**Sulfide-quinone reductase**					

AFE_1792	*sqr*	Sulfide-quinone reductase	-0,1	≈	CCM 4253/[24] NASF-1/[26]

**Others**					

AFE_0049	-	periplasmic solute-binding protein, putative	0,3	≈	ATCC 23270/[29]

AFE_1663	*glcF*	glycolate oxidase, iron-sulfur subunit	-1,7	S	

AFE_1677	*cbbOIa*	von Willebrand factor type A domain protein	-1,9	S	

AFE_2971	*cysN2*	sulfate adenylyltransferase, large subunit	-1,3	S	

AFE_0282	*fur*	ferric uptake regulator	0,6	≈	

AFE_2324	*pgm*	phosphoglucomutase	0,6	≈	

AFE_0445	*galU*	UTP-glucose-1-phosphate uridylyltransferase	0,2	≈	

AFE_1342	*epsS*	UDP-glucose 4-epimerase	0,0	≈	

AFE_2840	*malQ*	glycosyl hydrolase	-0,2	≈	

AFE_2836	*glbB*	1,4-alpha-glucan branching enzyme	-0,6	≈	

AFE_3054	*cbbOIb*	von Willebrand factor type A domain protein	-0,1	≈	

AFE_2157	*cbbOII*	von Willebrand factor type A domain protein	-0,6	≈	

AFE_0539	*cysN3*	sulfate adenylyltransferase, large subunit	0,1	≈	

AFE_2602	-	hypothetical	-0,1	≈	ATCC 19859/[25]

Real time PCR gene expression values (log_2 _ratio of median) for relevant genes alluded in the revised iron or sulfur oxidation models for *A. ferrooxidans *ATCC 23270. Genes with a log_2 _ratio of median larger than |1.5| (corresponding to genes induced more than 2.8 fold) are considered differentially expressed (indicated with *). Gene ID is that of Genbank genome annotation NC 011761. The reference and the strain in which the level of the gene product has been shown to be higher in one of the conditions tested (Fe(II) or S^0^) are indicated in the last column.

### Extending the current model of Fe(II) oxidation

The *rus *operon was induced in Fe(II)-grown cells (Table 1[7,24,25] and Table 3[7,24-26,28,29]) supporting the current model of the involvement of Cyc2, rusticyanin, Cyc1 and cytochrome oxidase in the oxidation of Fe(II) and the downhill electron transfer chain terminating in the reduction of oxygen to water (Figure 1).

Embedded in the *rus *gene operon, is a hypothetical gene of unknown function (*ORF1*, AFE_3151). Its genetic linkage and congruent transcriptional activity suggest that it is involved in Fe(II) oxidation (Table 1[7,24,25] and Table 3[7,24-26,28,29]). In agreement with these data, highest expression of ORF1 was detected in iron-compared to sulfur-grown ATCC 33020 cells [7]. ORF1 exhibits weak similarity to the putative type-3 multicopper oxidase from *Halorubrum lacusprofundi *(30% similarity, e-value: 8e-05) and to the predicted outer membrane type 3 multicopper oxidase protein Pan1 from *Halobacterium *sp. NRC-1 (32% similarity; e-value: 3e-04). It also exhibits weak similarity to rusticyanin including conservation of three out of its four critical copper binding residues. In addition, EPR analysis suggests that ORF1 contains copper [30]. We propose the name Cup (cupredoxin-like) for ORF1. A possible function for Cup is to deliver copper either to *aa*_3 _cytochrome oxidase or to rusticyanin. The well documented proteins Sco and Cox1 that are involved in copper delivery to copper-containing proteins in other organisms [31] have been not detected in *A. ferrooxidans *[8] and Cup may have assumed their role. Given its similarity to rusticyanin, an alternate hypothesis is that Cup is involved in electron transfer perhaps between Cyc2 and Cyc1 bypassing rusticyanin. Two arguments in favor of this hypothesis are: 1) Cup has been shown experimentally to be physically associated with Cyc1 and not with rusticyanin [15], and 2) Cup is bound to the outer membrane likely facing the periplasm [Amouric, Yarzabal and Bonnefoy, unpublished results]. Cup could provide an alternative route for electron flow during iron oxidation and an additional point for its regulation.

Immediately downstream of the *rus *operon is a cluster of six genes, upregulated in iron (Table 1[7,24,25] and Table 3[7,24-26,28,29]), that are predicted to be involved in cytochrome *aa*_3 _oxidase biogenesis (*ctaABT*) and iron-responsive regulation of cytochrome *aa*_3 _oxidase biogenesis (*ctaRUS*): *ctaA *(AFE_3144) encoding an integral membrane protein with 7 out of 8 His located in transmembrane regions similar to heme A synthase CtaA ([32] and references therein), *ctaB *(AFE_3143) encoding a heme O synthase and *ctaT *(AFE_3142) encoding an integral membrane protein belonging to the major facilitator family transporter that could be involved in the exportation of heme A to cytochrome oxidase [33] (Figure 1); *ctaR *(AFE_3141) predicted to encode an iron responsive regulator [34] of the Rrf2 family that in *T. denitrificans *is clustered with *cbb*_3 _cytochrome oxidase biogenesis genes (data not shown), *ctaU *(AFE_3139) encoding a hypothetical protein of unknown function and *ctaS *(AFE_3138) encoding a predicted Fe(II)-dependent oxygenase superfamily member of unknown function. Immediately downstream but transcribed in the other direction, are two genes *regBA *(AFE_3136-3137), also upregulated in iron (Table 1[7,24,25] and Table 3[7,24-26,28,29]), that are predicted to encode a sensor/regulator two-component signal transduction system of the RegB/RegA family with similarity to RegBA of *Rhodobacter capsulatus*. RegA directly controls synthesis of cytochrome *cbb*_3 _and ubiquinol oxidases that function as terminal electron acceptors in a branched respiratory chain [35]. Given the sequence similarity of the predicted *A. ferrooxidans *RegA with that of *R. capsulatus*, the conservation of the quinone binding site in the membrane spanning domain and of the redox active cysteine in the cytoplasmic transmitter domain of RegB, we propose that it is also involved in redox sensing. Because of the *regBA *juxtaposition to genes predicted to encode cytochrome *aa*_3 _oxidase biogenesis, this cytochrome oxidase is a likely candidate for the target of RegBA regulation in *A. ferrooxidans*. However, in *R. capsulatus*, RegBA also regulates other genes involved in respiratory electron components such as cytochromes *c*_2_, c(y) and the cytochrome *bc*_1 _complex, so that the actual RegBA target(s) in *A. ferrooxidans *requires experimental evaluation. *RegBA *of *R. capsulatus *have been shown to respond to the status of the aerobic respiratory chain, most likely the ubiquinone pool in the membrane [36] and, if this also proves to be the case in *A. ferrooxidans*, RegBA could help in making regulatory changes to balance electron equivalents between uphill and downhill electron flow, perhaps by adjusting the proportion of cytochrome Cyc1 and cytochrome oxidase *aa*_3 _(downhill electron flow) to the cytochrome CycA1 and the cytochrome *bc*_1 _complex (uphill electron flow). Alternately, RegBA could play a role in switching between iron and sulfur oxidation or between aerobic and anaerobic oxidation. It is clear that the discovery of the predicted iron and redox responsive regulators CtaRUS and RegAB will now allow the experimental biologist to focus on important regulatory switches that are most likely to be involved in cellular decisions related to energy metabolism.

The *petI *operon was also induced in Fe(II) medium (Table 1[7,24,25] and Table 3[7,24-26,28,29]) supporting the current model for the role of the *bc*_1 _complex in the uphill flow of electrons during iron oxidation (Figure 1). Embedded within the *petI *operon is *sdrA1 *(AFE_3108) whose function remains unknown. SdrA1 has the characteristic NAD(P) binding site at its N-terminus (TGAGEGIG) of Ndu9 which is a subunit of the NADH complex involved in this complex assembly and stability in a variety of eukaryotes [37]. However, SdrA1 exhibits the conserved catalytic residues (N125, S147, Y166, K170) involved in electron input into the NADH complex or electron transfer within the complex, suggesting that this protein could function as an oxidoreductase [38,39] (Additional file 2). SdrA1 has been predicted to be situated in the cytoplasm in *A. ferrooxidans *with a possible hydrophobic region embedded in the inner membrane [12] and we hypothesize that it transfers electrons from the quinone pool to the NADH complex (Figure 1).

Whereas most of the predicted genes encoding the subunits of the NADH complex (AFE_2630-2617) are equally expressed in Fe(II) and S^0 ^growth conditions, *nuoI *(AFE_2622) and *nuoK *(AFE_2620) are upregulated in Fe(II) medium (Table 1[7,24,25]). The *nuoI *encodes a ferredoxin located in the cytoplasmic arm of the NADH complex and is involved in the intramolecular electron transfer between FMN and quinone, whereas *nuoK *encodes a membrane subunit thought to be involved in quinone reduction [40] and in proton translocation [41]. Given the predicted locations of NuoI and NuoK in the hinge region of the NADH complex [42] and the upregulation of *nuoI*, *nuoK *and *sdrA1 *in Fe(II) growth conditions, we suggest that they interact to facilitate uphill electron flow from quinone to the NADH complex (Figure 1).

Whereas most of the genes predicted to encode the ATP synthetase complex were similarly expressed in Fe(II) and in S^0 ^grown cells, membrane embedded F0 subunits A and C, encoded by *atpB *(AFE_3209) and *atpE *(AFE_3208) respectively, were upregulated in Fe(II) growth conditions (Table 1[7,24,25]). These subunits are involved in proton translocation across the membrane [43] and their upregulation could allow more protons to pass through the ATP synthetase complex during Fe(II) oxidation, provided an increase in ATP synthesis. However, the resulting increase of intracellular protons requires a concomitant increase in intracellular electrons for their neutralization, so as not to compromise the internal pH of the cell. These electrons could come from the downhill pathway during Fe(II) oxidation as shown in Figure 1.

The organization and regulation of the components of Fe(II) oxidation in *A. ferrooxidans *appear to be unique to this organism, although *Thiobacillus prosperus *strain V6 possesses a transcriptional unit upregulated in iron conditions with some similarity to the *rus *operon [44]. However, the *T. prosperus *operon lacks the *cyc1 *and the *rus *genes encoding cytochrome *c*_4 _and rusticyanin, respectively. The latter is located downstream from the cluster, is monocistonic and is expressed in iron and sulfur growth conditions [44], suggesting different regulatory mechanisms compared to *A. ferrooxidans*. In *Leptospirillum *group II, the electron transfer chain involved in Fe(II) oxidation contains two cytochromes *c *and a *cbb*_3 _cytochrome oxidase [45-47] but no blue copper protein such as rusticyanin. In the archaea *Ferroplasma acidarmanus *a blue copper protein (sulfocyanin) has been suggested to transfer the electrons from Fe(II) to a *cbb*_3_-type terminal oxidase [48]. While the genes encoding four blue copper proteins (two sulfocyanin-like and two rusticyanin-like) have been identified in *Metallosphaera sedula *[49], none of them respond to the presence of iron in the medium [50]. However, *foxA *(cytochrome oxidase subunit I) and *soxNL *(cytochrome *b *and [2Fe-2S] Rieske) -*cbsAB *(cytochromes *b*) clusters have been predicted to be important for Fe(II) oxidation in *M. sedula*. Genes encoding cytochrome *c *oxidase subunits I and II (*foxAB*) and CbsA-like cytochrome *b *(*foxC*) have been also proposed to be involved in iron oxidation in *Sulfolobus metallicus *[51]. It appears therefore that different pathways for ferrous iron oxidation have evolved in prokaryotes.

### Extending the current model of the oxidation of reduced inorganic sulfur compounds (RISCs)

**Figure 2:**
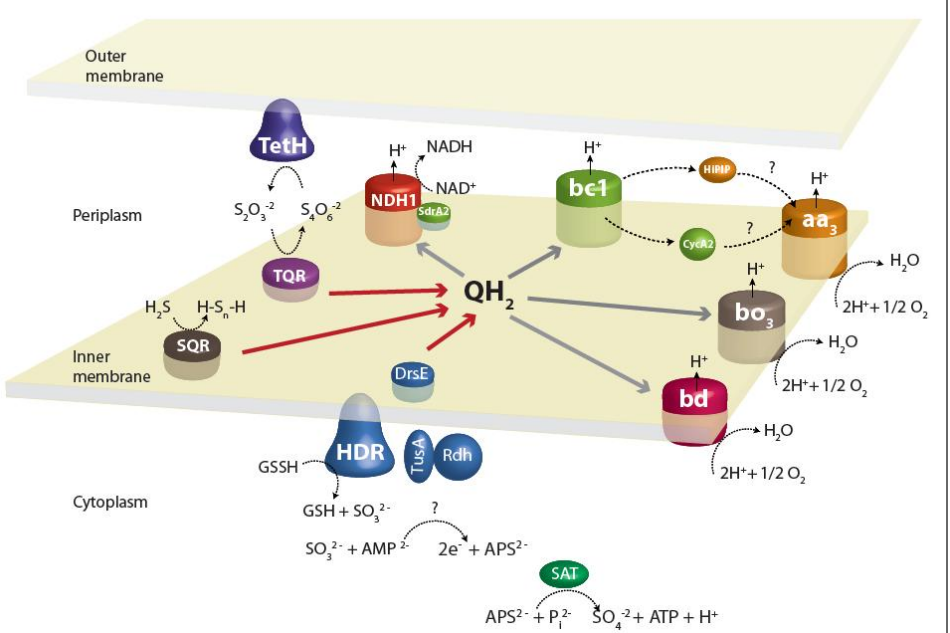
**Model of sulfur oxidation in *A. ferrooxidans *ATCC 23270**. Reduced inorganic sulfur compound (RISC) oxidation pathways are predicted to involve various enzymes, enzyme complexes and a number of electron carriers located in different cellular compartments: in the outer membrane facing the periplasm (tetrathionate reductase, TetH), in the periplasm (high potential iron-sulfur protein, HiPIP), attached to the cytoplasmic membrane on the periplasmic side (cytochrome *c*, CycA2), in the cytoplasmic membrane (sulfide quinone reductase (SQR), thiosulfate quinone reductase (TQR), *bc*_1 _complex, NADH complex I, *bd *and *bo*_3 _terminal oxidases) and in the cytoplasm (heterodisulfide reductase (HDR), and ATP sulfurylase (SAT)). Insoluble sulfur is first converted to sulfane sulfate (GSSH) which is then transferred to the heterodisulfide reductase (HDR) through a cascade of sulfur transferases (DsrE, TusA and Rhd). Electrons coming from sulfide (H_2_S), thiosulfate (S_2_O_3_^2-^) or sulfane sulfate (GSSH) are transferred via the quinol pool (QH_2_) either (1) directly to terminal oxidases *bd *or *bo*_3_, or indirectly throught a *bc*_1 _complex and a cytochrome *c*(CycA2) or a high potential iron-sulfur protein (HiPIP) probably to the *aa*_3 _oxidase where O_2 _reduction takes place or (2) to NADH complex I to generate reducing power.

Predicted genes, proteins or enzymatic activities previously identified in the oxidation of RISCs in different species of *A. ferrooxidans *include: *sqr *(AFE_1792) encoding sulfide quinone reductase from NASF-1 strain [26,52], *doxDA *(AFE_0044) encoding thiosulfate quinone oxidoreductase from ATCC23270 [53] and from the CCM4253 strain [54], *tetH *(AFE_0029) encoding tetrathionate hydrolase from ATCC23270 [27], *cydAB *(AFE_0955-0954) encoding a *bd *oxidase and *cyoABCD *(AFE_0631-0634) encoding a *bo*_3 _oxidase from ATCC19859 [11] (See Figure 2). All these genes are upregulated in sulfur-containing media relatively to Fe(II) (Table 2[24-29] and Table 3[7,24-26,28,29]). This is also the case for the *petII *operon (AFE_2727-2732) encoding a second *bc*_1 _complex, a cytochrome *c*_4_, SdrA2 and a high potential iron-sulfur protein, Hip, as already reported for ATCC19859 [11], ATCC33020 [13] and ATCC23270 [14] strains (Table 2[24-29] and Table 3[7,24-26,28,29]). This second *bc*_1 _complex has been proposed to function directly transferring electrons from sulfur to oxygen (Figure 2), and possibly in the aerobic and anaerobic oxidation of sulfur and formate described by Pronk *et al*. [55]. In that case, the *bc*_1 _complex receives the electrons from the quinol pool and transfers them to the membrane-bound cytochrome *c*_4 _CycA2 and/or to the periplasmic high potential iron-sulfur protein Hip that subsequently gives the electrons to the terminal oxidase [2,13,14] (Figure 2). SdrA2, like SdrA1 (see above), may promote electron flow from the quinone pool to the NADH complex (Figure 2). These findings support earlier models of the branched electron transfer flow during S^0 ^oxidation [2,11,13,14] (Figure 2). However, this model is far from being complete and several outstanding questions remain unanswered, including the identification of the enzymes that oxidize sulfur and sulfite.

### Sulfur

Seven genes, potentially encoding a heterodisulfide reductase complex HdrABC (AFE_2586 and AFE_2555-2550) were highly upregulated in cells grown in sulfur medium (Table 2[24-29] and Table 3[7,24-26,28,29]). This complex catalyzes the reversible reduction of the disulfide bond X-S-S-X coupled with energy conservation in methanogenic archaea ([56] and references therein) and sulfate reducing archaea and bacteria [57]. This complex in *A. ferrooxidans *is predicted to have three different cytoplasmic HdrB subunits (AFE_2586, 2554 and 2550) with the cysteine-rich domain which binds the unusual type [4Fe-4S] cluster [58], involved in disulfide reduction [56,59]. In addition, genes potentially encoding the ferredoxin-like and the flavoprotein subunits, HdrC (AFE_2555 and 2551) and HdrA (AFE_2553) are present in the same locus. The flavoprotein HdrA subunit exhibits a possible N-terminal membrane spanning region, a conserved FAD binding site (GXGXXGX_16–19_(D/E)), and the conserved four cysteine cluster (CXGXRDX_6–8_CSX_2_CC) that binds a Fe-S center, typical of the cytoplasmic iron-sulfur flavoprotein type enzyme.

Three genes encoding for sulfur metabolism accessory proteins, placed immediately upstream of the heterodisulfide reductase complex, are similarly upregulated (Table 2[24-29]). These encode a cytoplasmic rhodanase-related sulfurtransferase (AFE_2558, COG0607) [60], a cytoplasmic SirA-like disulfide bond formation regulator (AFE_2557, pfam01206, COG0425, IPR001455) and an inner membrane located peroxiredoxin of the DrsE superfamily (AFE_2556, COG2210 and 2044). The rhodanase AFE_2558 is 48% similar to the Sud protein from *Wolinella succinogenes *which binds and transfers polysulfide sulfur to the polysulfide reductase located in the cytoplasmic membrane [61,62]. In turn, the SirA-like protein AFE_2557 is 54% similar to the TusA protein from *Vibrio fischeri *and other bacteria which belong to a complex sulfur-relay system that facilitates specific sulfur flow/trafficking from various pathways [63-65]. Finally, DsrE family proteins such as that predicted to be encoded by AFE_2556 are small proteins recently shown to be involved in sulfur transfer reactions during sulfur oxidation [66].

**Figure 3:**
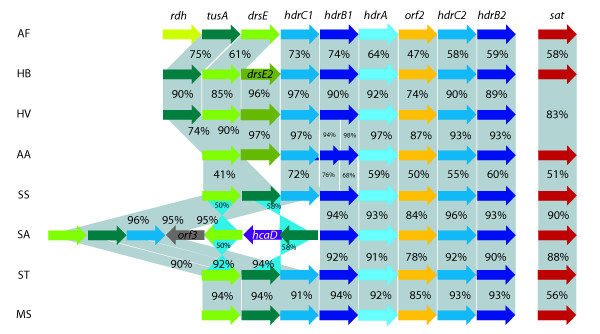
**Comparison of the *hdr *cluster between *A. ferrooxidans *ATCC 23270 and other sulfur oxidizers**. Heterodisulfide reductase complex (HdrC_1_B_1_AOrf2HdrC_2_B_2_), accessory proteins (Rhd, TusA, DsrE) and ATP sulfurylase (Sat) in AF: *A. ferrooxidans *ATCC 23270 (NC_011206), AA: *Aquifex aeolicus *(NC_000918) and known acidophilic sulfur oxidizing microorganisms HB: *Hydrogenobaculum *sp. Y04AAS1 (NC_011126), HV: *Hydrogenivirga *sp. 128-5-R1-1 (NZ_ABHJ00000000), MS: *Metallosphaera sedula *(NC_009440), SA: *Sulfolobus acidocaldarius *(NC_007181), ST: *S. tokodaii *(NC_003106) and SS: *S. solfataricus *(NC_002754). Percentage of amino-acid similarity is indicated. Blue triangles represent inversion in the gene order.

Significant sequence similarity and conserved gene organization of the *A. ferrooxidans *heterodisulfide reductase complex and accessory proteins is restricted only to *Aquifex aeolicus *and known acidophilic sulfur oxidizing microorganisms *Hydrogenobaculum *sp. Y04AAS1, *Hydrogenivirga *sp. 128-5-R1-1, *Metallosphaera sedula *[50], *Sulfolobus acidocaldarius*, *S. tokodaii *and *S. solfataricus *(Figure 3), associating the whole gene cluster with sulfur oxidation. In methanogenic and sulfate reducing archaea, HdrA receives the electrons from the hydrogenase and transfers them through HdrC to the heterodisulfide reductase catalytic site located in HdrB. Accompanying the reduction of heterodisulfide, protons are extruded across the membrane creating a proton motive force. We hypothesize that in *A. ferrooxidans *and the sulfur oxidizers referred above, the Hdr complex, driven by the naturally existing proton gradient, could be working in reverse and oxidizing disulfide intermediaries (from sulfur oxidation) to sulfite and delivering the collected electrons to the membrane quinol pool (Figure 2). In addition, the three accessory sulfurtransferases are likely involved in the transfer of the proposed sulfane sulfur [67,68] to the heterodisulfide reductase (Figure 2).

The predicted *A. ferrooxidans *heterodisulfide reductase complex has additional features that are consistent with its proposed role in RISCs oxidation. Sulfur oxidation in a variety of different *A. ferrooxidans *strains has been shown to require glutathione [67,68] and requires a neutral pH optimum [67], suggesting a cytoplasmic activity in agreement with the predicted localization of the heterodisulfide reductase catalytic site (Figure 2). Second, non-heme iron and labile sulfur have been shown to be present in a sulfur oxidizing enzyme preparation of *A. ferrooxidans *[67] and iron-sulfur clusters are predicted to be present in the *A. ferrooxidans *HdrB and C subunits. Third, sulfur oxidation has been shown to be inhibited by HQNO in *A. ferrooxidans *[69], in agreement with the proposal that the quinone pool is the physiological electron acceptor (Figure 2). Fourth, the actual substrate of the sulfur oxidizing enzyme in *A. ferrooxidans *is thought not to be elemental sulfur, which has poor water solubility and cannot enter the cell, but rather sulfane sulfur of GS_n_H species (*n*>1) and most likely GSSH [67,68]. Sulfane sulfate would thus provide the necessary disulfide bond X-S-S-X to serve as a substrate for the predicted catalytic activity of the *A. ferrooxidans *heterodisulfide reductase (Figure 2). Conserved within the heterodisufide reductase gene cluster, between *hdrA *and *hdrC2*, is a putative gene of unknown function (AFE_2552) whose product is predicted to reside in the cytoplasm (Figure 2). Given its conserved gene context, we propose that it is also involved in RISCs oxidation and it is now pinpointed for experimental investigation.

### Sulfite

Another step in the sulfur oxidation model that awaits genetic characterization is sulfite oxidation. Being metastable and considered short-lived in mine waste environments, one possibility is that sulfite rapidly oxidizes non-enzymically to sulfate, thiosulfate or glutathione S-sulfonate in the presence of Fe(III) [70,71] or sulfur [72]. However, the involvement of a protein catalyzing this reaction is more likely since a sulfite oxidase activity was purified from three different strains of *A. ferrooxidans*, namely TM [73], ATCC13661 [74] and AP19-3 [75,76]

Genes coding for known periplasmic enzymes involved in the direct oxidation of sulfite during sulfur dissimilatory metabolism (*sorAB *or *soxCD *[77]) have not been detected in the *A. ferrooxidans *genome. In our model (Figure 2), sulfite is hypothesized to be produced in the cytoplasm by heterodisulfide reductase. Therefore, subsequent oxidation of sulfite is likely to occur in this cellular location and therefore would not be expected to proceed via the classical periplasmic Sor or Sox. One possibility for the cytoplasmic activity is that sulfite is converted to adenosine-5'-phosphosulfate (APS) via the well characterized APS reductase complex encoded by *aprBA *[78-80]. However, the genome contains no candidates with significant similarity to *aprBA*, although it does have a predicted *sat *(AFE_0539) which, in other microorganisms, encodes an ATP sulfurylase responsible for the second step in this pathway. The *A. ferrooxidans *Sat shares 44% identity and 60% similarity with both domains of the bifunctional SAT/APS kinase from *Aquifex aeolicus *that catalyzes the production of ATP and sulfate from APS and pyrophosphate [81,82]. If Sat is indeed catalyzing APS to sulfate (Figure 2), an enzyme catalyzing the oxidation of sulfite to APS is required. This missing function could be accomplished by the conserved hypothetical gene embedded in the *hdr *locus of sulfur oxidizers (Figure 3). The concordance of gene occurrence and organization between *A. aeolicus*, *Hydrogenobaculum *sp. Y04AAS1 the Sulfolobales and *A. ferrooxidans *including 1) the *hdr *locus with a gene of unknown function 2) *sat *and 3) a lack of *aprBA*, strongly suggests that these microorganisms have a novel sulfur oxidation pathway.

Our data agree with the model proposed for S^0 ^oxidation in *M. sedula*, including a heterodisulfide reductase (*hdr*), a tetrathionate hydrolase (*tetH*), a terminal oxidase complex based on both quinol oxidase (*soxCL*) and *aa*_3_-type cytochrome oxidase (*soxAB*) components [50]. In *S. metallicus*, a gene (*sor*), encoding sulfur oxygenase reductase, which is absent in *A. ferrooxidans*, is the dominant transcript in sulfur-grown cells and is therefore proposed to be involved in sulfur oxidation [51]. In conclusion, the RISCs oxidation pathways of acidophiles are not only different from those of neutrophilic sulfur oxidizers [17,18] but also appear to be different among the acidophilic sulfur-oxidizers including between members of the *Acidithiobacillus *genus [83]. In that sense, the conservation of the *hdr *locus in different acidophilic sulfur oxidizers, archaea and eubacteria, is noteworthy and merits further investigation.

### Additional Discussion

Genomic and transcriptomic (microarrays and real-time quantitative PCR) studies of iron and sulfur energetic metabolism of *A. ferrooxidans*, not only confirm previous data, but elaborate on the complexity of these pathways. Both iron and RISCs respiratory chains are branched and redundant [2,11,13,14] (Figures 1 and 2). This provides *A. ferrooxidans *with a flexible respiratory system that may allow it to adapt efficiently to environmental changes by modulating gene expression according to the growth conditions (substrate, oxygen concentration, growth phase, etc.). Another way to adapt efficiently to a change in the growth conditions is by modifying the association of complexes as suggested recently for iron respiration in *A. ferrooxidans *[15]. In the case of the cytochrome *c *oxidase complex of *Dictyostelium discoideum*, the oxygen concentration induces a switch between two interchangeable subunit isoforms of the cytochrome *c *oxidase [84-86]. This switch has been shown to be due to transcriptional regulation and also to different stabilities of the two subunits toward oxygen [86].

Both iron and RISCs oxidation pathways involve outer membrane, periplasmic and inner membrane components (Figures 1 and 2). According to our hypothesis, several super-complexes spanning the outer and/or the inner membranes are expected to conduct either the electrons to the oxygen, or the sulfane-sulfur to the catalytic side of the herodisulfide reductase, from pyrite (FeS_2_), which is a natural substrate of *A. ferrooxidans*. While such a super-complex has been isolated recently for iron oxidation [15], no biochemical data are available until now to substantiate the existence of a sulfane-sulfur transfer supramolecular structure. While a sulfurtransferase complex encoded by the *hdr *locus is likely to be involved in the transfer of the proposed sulfane sulfur to the heterodisulfide reductase from the inner membrane to the cytoplasm (Figure 2), expression data suggests no obvious upregulated outer membrane protein as proposed by Rohwerder and Sand [68] to allow it to cross the cell wall. We propose also the existence of a cytoplasmic super-complex catalyzing both the oxidation of sulfane-sulfur to sulfite and of sulfite to APS, preventing the accumulation of sulfite in the cytoplasm (Figure 2). While the existence of such a complex has not been demonstrated in *A. ferrooxidans*, a thiosulfate-oxidizing system oxidizing hydrogen sulfide, thiosulfate, sulfur and sulfite directly to sulfate without the presence of free intermediates has been evidenced in *Paracoccus versutus *and *Paracoccus pantotrophus *([17,87] and references therein). Moreover, the methanogenic and sulfate reducing archaea heterodisulfide reductase forms a tight complex with the hydrogenase, which catalyzes its reduction with H_2 _([56,57] and references therein). Such supramolecular structures will allow (1) stabilization of the different components (2) electron, or sulfane sulfur, channeling leading to more efficient transfer, and (3) diffusion avoidance preventing toxic compound leakage.

## Conclusion

• Bioinformatic analysis coupled with gene transcript profiling extends our understanding of the iron and reduced inorganic sulfur compounds oxidation pathways in *A. ferrooxidans*.

• Novel genes predicted to be involved in iron oxidation (Figure 1) include those potentially encoding: i) heme biosynthesis and insertion into the terminal electron acceptor cytochrome *aa*_3 _oxidase used in the downhill flow of electrons from Fe(II) to reduce oxygen to water, ii) subunits of the F_o _ATP synthetase that may be an adaptation to extremely low pHs encountered during iron oxidation, iii) a copper-containing protein that may be involved in electron transfer or in copper insertion, iv) two subunits and one predicted accessory protein of the NADH complex that may promote the flow of electrons from the quinone pool to the NADH complex during uphill electron flow and v) two potential regulators of alternate electron pathways predicted to respond to iron and the status of the quinone pool respectively.

• Novel genes predicted to be involved in RISCs oxidation (Figure 2) include those potentially encoding: i) three sulfurtransferases, ii) a heterodisulfide reductase complex, iii) an ATP sulfurylase and iv) a NADH complex accessory protein that may promote uphill electron flow from the quinone pool to the NADH complex during RISCs oxidation.

• The models (Figures 1 and 2) provide unified and coherent descriptions of iron and RISCs oxidation and suggests mechanisms for their regulation within the type strain, eliminating previous confusion caused by models built from analyses of multiple and divergent strains of this microorganism.

• The identification of differentially expressed genes of unknown function predicted to be involved in iron and RISCs oxidation direct the experimental biologist to future research.

## Methods

### Strains and culture conditions

*A. ferrooxidans *type strain ATCC 23270 was obtained from the American Type Culture Collection. *A. ferrooxidans *was grown at 30°C under oxic conditions with 200 rpm agitation in (i) Fe(II)-medium (62 mM FeSO_4_-7H_2_O in basal salts solution ((NH_4_)_2_SO_4_: 0.4 g. L^-1^, K_2_HPO_4_: 0.4 g. L^-1^, MgSO_4_-7H_2_O: 0.4 g. L^-1 ^adjusted to pH 1.6 with H_2_SO_4 _or (ii) S^0^-medium (1% (w/v) elemental sulfur in basal salts solution ((NH_4_)_2_SO_4_: 0.4 g. L^-1^, K_2_HPO_4_: 0.4 g. L^-1^, MgSO_4_-7H_2_O: 0.4 g. L^-1 ^adjusted to pH 3.5 with H_2_SO_4_). When reaching mid-logarithmic phase cells were harvested by centrifugation at 4°C and washed with basal salt solution (pH adjusted). To remove iron traces or sulfur particles before further treatment several cycles of washing and 1 minute low speed spin-centrifugations were performed. Clean cell pellets were frozen in liquid nitrogen and stored at -80°C until used for RNA extraction.

### RNA isolation

Total RNA was extracted using a modified acid-phenol extraction method [88], including a preliminary TRIZOL^® ^reagent (Invitrogen) extraction step. This RNA was used directly for cDNA synthesis and labeling. For real-time PCR, RNAs were additionally purified with the High Pure RNA isolation kit (Roche Applied Biosystem) and treated twice with DNAse I (Roche Applied Biosystem). The lack of DNA contamination was checked by PCR on each RNA sample.

### Microarray production

*A. ferrooxidans *ATCC 23270 oligonucleotide microarrays were designed and produced in-house. Internal 50-mers targeting each of the 3037 putative ORFs predicted in the genomic sequence of *A. ferrooxidans *ATCC 23270 (TIGR release September 2003; 2.98 Mb) were selected using the Oligoarray software (Version 1.0), synthesized by MWG Biotech and spotted in duplicate on Gamma Amino Propyl Silane Corning UltraGAPS slides, according to the manufacturer's instructions, with a ChipWriterProarrayer (Bio-Rad, 1000 Alfred Nobel Drive Hercules, CA) at the Marseille-Nice Genopole (France). A special set of control oligonucleotides (negative, positive, tiled and antisense sequences) was included to evaluate probe specificity and adjust the hybridization conditions. 50-mer oligonucleotides corresponding to the CDS of the *gfp *gene from *Aequorea *sp. (L29345), the *rad9 *gene form *Saccharomyces cereviciae *(M26049) and the *idi2 *gene from human (AK303669) were used as negative controls. The array design and spotting protocol were deposited in ArrayExpress database [89] under the accession codes A-MEXP-1478 and A-MEXP-1479.

### Microarray experimental design

This experiment consisted of 8 independent hybridizations using total RNA obtained from 2 different iron- (FeIIA and FeIIB) and 2 different sulfur-replicate cultures (S°A and S°B). Each RNA sample was labeled once with each dye (e.g. S^0 ^A_Cy^®^5 and S^0 ^A_Cy^®^3) and hybridized against a reciprocally labeled cDNA sample (e.g. FeIIA_Cy^®^3 and FeIIA_Cy^®^5). The cDNAs obtained for each biological replicate (e.i. A or B) were cohybridized against two different types of microarrays obtained in different spotting campaigns (Oligoarray v.1 or Oligoarray v.2). More details of the experimental design are available in the ArrayExpress database under the accession code E-MEXP-1990.

### Labeling and hybridization

The ChipShot™ Labeling Clean-Up System (Promega-Corning) was used to generate fluorescently labeled cDNA via direct incorporation of Cy^®^3 and Cy^®^5-labeled nucleotides (Amersham Biosciences). The reverse transcription reaction was performed in the presence of 5 μg total RNA and random hexamers according to manufacturer's recommendations. Two independent cDNA preparations were labeled once with each dye (reverse dye labeling) to account for sampling differences, biases in dye coupling or emission efficiency of Cy^®^-dyes. Labeled cDNA was purified from contaminating fluorescent dNTPs and degraded RNA using the ChipShot Labeling Clean-Up System (Promega-Corning). Dye incorporation efficiency was determined by absorbance readings at 260, 550 and 650 nm and the frequency of incorporation (FOI, pmol of dye incorporated per ng of cDNA) was calculated according to Promega's instructions. Optimally labeled samples were combined, vacuum-dried and resuspended to a final volume of 40–50 μL in Corning Pronto! Universal Hybridization Buffer. The combined denatured target cDNA samples (95°C for 5 min) were hybridized to the spotted slides for 14 h at 42°C in the Corning hybridization chamber. Following hybridization, slides were washed in serial dilutions of Corning Wash Buffers as recommended by the manufacturer and dried by centrifugation at 1200 × *g *for 2 min.

### Image acquisition and data analysis

Microarrays were scanned for the Cy^®^3 and Cy^®^5 fluorescent signals using the ScanArray 5000 Microarray Analysis System (PerkinElmer Life Sciences, Inc.). Scans stored as 16-bit TIFF (Tagged Information File Format) image files and then analyzed with the image quantification software package GenePix Pro 6.0 (Axon Instruments, Inc.). Saturated and low-quality spots (spots smaller than 60 μm or bigger than 160 μm in diameter, sub-circular in shape and/or exhibiting uneven fluorescence distribution) were flagged and filtered out. Local background was subtracted from the recorded spot intensities and median values for each spot were Log transformed (log_2_) and normalized by average intensity of each slide to account for any difference in total intensity between the scanned images using the Acuity microarray analysis software (Version 4.0, Axon Instrument Inc.). The processed Fe(II) and S^0 ^signal intensities for each spot were used for calculating the expression ratio Fe(II)/S^0^. Twenty four replicate ratio values per gene, resulting from direct and reverse labeling, replicate spots and replicate experiments, were used for further statistical analysis of the data. The statistical significance of differential expression in Fe(II) or S^0 ^grown cells, was assessed using Acuity package. The raw data, as well as the processed (filtered and Median normalized) data, for all hybridizations was submitted to the ArrayExpress database and is available under the accession code E-MEXP-1990. A 1.5 fold deviations from the 1:1 hybridization ratio was taken as indicative of differential gene expression in the two growth conditions analyzed. Hierarchical cluster analysis (Pearson correlation, average linkage) was performed using Genesis software suit [90]. Functional annotations were retrieved from Valdés et al. [8].

### Real-time quantitative PCR

The relative abundances of a set of differentially expressed genes and a set of invariant genes, according to the microarray results obtained, were determined in Fe(II)- and S^0^-grown cells by real-time PCR. Specific primers for the genes of interest amplifying average products of 300 bp with about 50 GC% and about 55°C Tm were designed (Additional file 3). Equal amounts of *A. ferrooxidans *DNAseI-treated total RNA were retro-transcribed from these primers with the Superscript II™ RNase (Invitrogen Life Technologies) at 42°C for 50 min, and then treated at 70°C for 15 min to inactivate the enzyme. The real-time PCR quantifications were performed on the cDNA obtained, using the Light-Cycler instrument and the LightCycler Fast Start DNA master (plus) SYBR Green I PCR kit (Roche Applied Biosystems) with external standards as described in Roche Molecular Biochemicals technical note no. LC 11/2000. The real-time PCR experiments were performed twice, with both independent total RNA and cDNA preparations by the comparative threshold cycle method. The calculated threshold cycle (Ct) for each gene was normalized to Ct of the *rrs *gene.

### Bioinformatic analysis

The sequence and annotation of the complete *A. ferrooxidans *strain ATCC 23270 genome was retrieved from GenBank/EMBL/DDBJ (CP001219). Functional assignments for hypothetical and predicted genes of interest were performed manually on a gene-by-gene basis. Amino acid sequences for these genes were used to query the following databases: National Center for Biotechnology Information (NCBI) nonredundant database, UniProt, TIGRFam, Pfam, PRIAM, KEGG, COG and InterPro. Comparative genomic analyses were performed using the Comprehensive Microbial Resource [91], Microbesonline [92] and String [93].

## Authors' contributions

DH and EJ conceived the study; CA, RQ, DH and VB designed the experiments; CA and RQ carried out the experiments (each contributed equally); YD assisted in the microarray experiments; CA, RQ, DH and VB interpreted the results and wrote the paper. All authors read and approved the paper.

## Supplementary Material

Additional file 1 **Differentially expressed genes**. Genes with a log_2 _ratio of median greater than 1.5 (corresponding to genes induced more than 2.8 fold) were considered differentially expressed in the two growth conditions. Functional categories are those defined by the TIGR database [94]. (Quatrini, R., C. Appia-Ayme, Y. Denis, E. Jedlicki, D. S. Holmes and V. Bonnefoy, BMC Genomics). Click here for file

Additional file 2 **Alignment of SdrA with Ndu9 subunit of the NADH complex**. The predicted classical NADH binding site in SdrA is highlighted in grey and catalytic residues involved in electron transfer in black. NduA9 and SdrA protein sequences ID in the alignment are as following: human (sp|Q16795|), horse (sp|Q5R5S0|), bovine (sp|P34943|), mouse (sp|Q9DC69|), *Neurospora crassa *(sp|P25284|), *A. ferrooxidans *SdrA1 (AFE_3108), *A. ferrooxidans *SdrA2 (AFE_2728). (Quatrini, R., C. Appia-Ayme, Y. Denis, E. Jedlicki, D. S. Holmes and V. Bonnefoy, BMC Genomics). Click here for file

Additional file 3 **Q-PCR primers used in this study**. (Quatrini, R., C. Appia-Ayme, Y. Denis, E. Jedlicki, D. S. Holmes and V. Bonnefoy, BMC Genomics). Click here for file
