# Supplementary material for: Extending the models for iron and sulfur oxidation in the extreme Acidophile Acidithiobacillus ferrooxidans
Source: BMC Genomics. 2009 Aug 24;10:394. doi: 10.1186/1471-2164-10-394 (PMC2754497; doi:10.1186/1471-2164-10-394)
Supplement: Additional file 2 — Alignment of SdrA with Ndu9 subunit of the NADH complex. The predicted classical NADH binding site in SdrA is highlighted in grey and catalytic residues involved in electron transfer in black. NduA9 and SdrA protein sequences ID in the alignment are as following: human (sp|Q16795|), horse (sp|Q5R5S0|), bovine (sp|P34943|), mouse (sp|Q9DC69|), Neurospora crassa (sp|P25284|), A. ferrooxidans SdrA1 (AFE_3108), A. ferrooxidans SdrA2 (AFE_2728). (Quatrini, R., C. Appia-Ayme, Y. Denis, E. Jedlicki, D. S. Holmes and V. Bonnefoy, BMC Genomics). [file 1471-2164-10-394-S2.pdf]

```
sp|Q16795|NDUA9_HUMAN      MAAAAQSRVVRVLSMSRSAITAIATSVCHGPPCRQLHHALMPHGKGGRSS 50
sp|Q5R5S0|NDUA9_PONPY      MAAAAQSRVVRVLSMSRSAITAIATSVCHGPPRRQLHHALIPHGKGGRSS 50
sp|P34943|NDUA9_BOVIN      MAAAVHPRVVRVLPMSRSSVPALAASVFHSPPQRQLHHAVIPHGKGGRSS 50
sp|Q9DC69|NDUA9_MOUSE      MAAAVRFRVVRALPMSRPAITAAATSVFCGSSHRQLHHAVIPHGKGGRSS 50
sp|P25284|NDUA9_NEUCR      -MAPLTAAMRSTPRIIVSNAFGFQRRAISDVTITRTGKPIIRN-QGGRSS 48
sdrA1                      ---------------MYAVYPEVLMNNYVPV----------------DGL 19
sdrA2                      -------------------VSAMTENDYTKAK--------------GDGI 17
                                                                           .

sp|Q16795|NDUA9_HUMAN      VSGIVATVFGATGFLGRYVVNHLGRMGSQVIIPYRCDKYDIMHLRPMGDL 100
sp|Q5R5S0|NDUA9_PONPY      VSGIVATVFGATGFLGRYVVNHLGRMGSQVIIPYRCDTYDIMHLRPMGDL 100
sp|P34943|NDUA9_BOVIN      VSGIVATVFGATGFLGRYVVNHLGRMGSQVIVPHRCEPYDTMHLRPMGDL 100
sp|Q9DC69|NDUA9_MOUSE      VSGVVATVFGATGFLGRYVVNHLGRMGSQVIIPYRCDVYDIMHLRLMGDP 100
sp|P25284|NDUA9_NEUCR      LGGHTATVFGATGQLGRYIVNRLARQGCTVVIPFR-DEYNKRHLKVTGDL 97
sdrA1                      LNGRVIIVTGAGEGIGRCAAVEFARLGAEVVLLGRTQRKLEGVYDEIVDS 69
sdrA2                      LDGKTILVTGAGDGIGRAVAIEYAHQGATVVLLGKTKRNLEGVYDEITDY 67
                           :.* .  * **   :**  . . .: *. *:: :  .           *

sp|Q16795|NDUA9_HUMAN      GQLLFLEWDARDKDSIRRVVQHSNVVINLIGRDWETKNFDFEDVFVKIPQ 150
sp|Q5R5S0|NDUA9_PONPY      GQLLFLEWDARDKDSIRRVVQHSNVVINLIGRDWETRNFDFEDVFVKIPQ 150
sp|P34943|NDUA9_BOVIN      GQIIFMDWNGRDKDSIRRAVEHSSVVINLVGREWETQNFDFEDVFVKIPQ 150
sp|Q9DC69|NDUA9_MOUSE      GQLTFLEWDARDKDSIRKAVQHSNVVINLIGREWETRNFDFEDVFVNIPR 150
sp|P25284|NDUA9_NEUCR      GKVVMIEFDLRNTQSIEESVRHSDVVYNLIGRDYPTKNFSFEDVHIEGAE 147
sdrA1                      G--------------YKEPVIHPMDLLTAKGRDYQDFAQRLKESLGRLDG 105
sdrA2                      G--------------YAEPAILVVDLADPASDAFKTIGAAISSEFTQLNG 103
                           *                . .     :    .  :      :..   .

sp|Q16795|NDUA9_HUMAN      AIAQLSKEAGVEKFIHVSHLNANIKSSSRYLRNKAVGEKVVRDAFPEAII 200
sp|Q5R5S0|NDUA9_PONPY      AIAQLSKEAGVEKFIHVSHLNANIKSSSRYLRNKAVGEKVVRDAFPEAII 200
sp|P34943|NDUA9_BOVIN      AIAQVSKEAGVEKFIHISHLNADIKSSSKYLRSKAVGEKEVRETFPEATI 200
sp|Q9DC69|NDUA9_MOUSE      AIAQASKEAGVERFIHVSHLNASMKSSSKSLRSKAVGEKEVRSVFPEAII 200
sp|P25284|NDUA9_NEUCR      RIAEAVAKYDVDRFIHVSSYNADPNSECEFFATKARGEQVVRSIFPETTI 197
sdrA1                      ILHN---AAELDILTPIQYYDEELWESAMKVNVTAP-YLLTQACLPLLLA 151
sdrA2                      IVHN---AAELGMLTPLENYEGALWDHVFQVNVKSP-LLVTQQCLPLLKE 149
                            : :      :  :  :.  :    .    .  .:     .:  :*

sp|Q16795|NDUA9_HUMAN      VKPSDIFGREDRFLNSFASMHRFGPIPLGSLGWKTVKQPVYVVDVSKGIV 250
sp|Q5R5S0|NDUA9_PONPY      IKPSDIFGREDRFLNSFASMHRFGPTPLGSLGWKTVKQPVYVVDVSKGIV 250
sp|P34943|NDUA9_BOVIN      IKPAEIFGREDRFLNYFANIRWFGGVPLISLGKKTVKQPVYIVDVTKGII 250
sp|Q9DC69|NDUA9_MOUSE      IRPSDIFGREDRFLNHFANYRWFLAVPLVSLGFKTVKQPVYVADVSKGIV 250
sp|P25284|NDUA9_NEUCR      VRPAPMFGFEDRLLHKLASVKN----ILTSNGMQEKYNPVHVIDVGQALE 243
sdrA1                      SEDASIVFITDDCAREAKGYWGAYAVSKAAVQHLGLTLAIELQNTNVRVN 201
sdrA2                      APYASIIFTTDESGVKPKGYWGAYGVSKAAILHMARMWAIEYANTPIRVN 199
                              : :.   *       .          :        .:   :.   :

sp|Q16795|NDUA9_HUMAN      NAVKDPDANGKSFAFVGPSRYLLFHLVKYIFAVAHRLFLPFPLPLFAYRW 300
sp|Q5R5S0|NDUA9_PONPY      NAVKDPDANGKSFAFVGPNRYLLFHLVKYIFAVAHRLFLPFPLPLFAYRW 300
sp|P34943|NDUA9_BOVIN      NAIKDPDARGKTFAFVGPSRYLLFDLVQYVFAVAHRPFLPYPLPHFAYRW 300
sp|Q9DC69|NDUA9_MOUSE      NATKDPDAVGKTFAFTGPNRYLLFHLVKYIFGMTHRTFIPYPLPLFVYSW 300
sp|P25284|NDUA9_NEUCR      QMLWDDNTASETFELYGPKTYTTAEISEMVDREIYKRRRHVNVPKKILKP 293
sdrA1                      VINPGPCRTG-----MRVRTHPGASIMSVPPPIAIMPLYEYLLGPDSKNT 246
sdrA2                      IVDPGPCRTG-----LRLLTHPGMPMKRYTPPEAITSIYTQLMDCDVLGH 244
                               .    .          :    :                :

sp|Q16795|NDUA9_HUMAN      VARVFEISPFEPWITRDKVERMHITDMKLPHLPGLEDLGIQATPLELKAI 350
sp|Q5R5S0|NDUA9_PONPY      VARVFEISPFEPWITRDKVERMHITDMKLPHLPGLEDLGIQATPLELKAI 350
sp|P34943|NDUA9_BOVIN      IGRLFEISPFEPWTTRDKVERIHTTDKILPHLPGLEDLGVEATPLELKAI 350
sp|Q9DC69|NDUA9_MOUSE      IGKLFGLSPFEPWTTKDKVVERIHISDVMPTDLPGLEDLGVQPTPLELKSI 350
sp|P25284|NDUA9_NEUCR      IAGVLNKALWWPIMSADEIEREFHDQVIDPEAKTFKDLGIEPADIANFTY 343
sdrA1                      RGQIMNARDWLDAEHDDRQIVAVLEQ------------------------ 272
sdrA2                      NGELFYAQNFINPDLDDRTEKDLATSTV---------------------- 272
                            . ::    :      *.       .

sp|Q16795|NDUA9_HUMAN      EVLRRHRT--YRWLSAEIEDVKPAKTVNI--- 377
sp|Q5R5S0|NDUA9_PONPY      EVLRRHRT--YRWLSAEIEDVKPAKTVNI--- 377
sp|P34943|NDUA9_BOVIN      EVLRRHRT--YRWLSSEIEDVQPAKTIPTSGP 380
sp|Q9DC69|NDUA9_MOUSE      EVLRRHRT--YRWLSSEIEETKPAKTVNY--- 377
sp|P25284|NDUA9_NEUCR      HYLQSYRSNAYYDLPPATEKERREDREYIHML 375
```
