# Supplementary material for: Extending the models for iron and sulfur oxidation in the extreme Acidophile Acidithiobacillus ferrooxidans
Source: BMC Genomics. 2009 Aug 24;10:394. doi: 10.1186/1471-2164-10-394 (PMC2754497; doi:10.1186/1471-2164-10-394)
Supplement: Additional file 3 — Q-PCR primers used in this study. (Quatrini, R., C. Appia-Ayme, Y. Denis, E. Jedlicki, D. S. Holmes and V. Bonnefoy, BMC Genomics). [file 1471-2164-10-394-S3.pdf]

NCBI Genome ID NC011761	Gene (locus)	Forward primer (5'-3')	Reverse primer (5'-3')	Amplicon size
***rus* operon**				
AFE_3151	*cup*	ATTGCGCATCAGTTCTCCAT	GGCACCACAAAACTGAAGGT	200
AFE_3146	*rus*	GATGGCCGGTACTCTGGATA	ATCTCCAAGGTCGGGTTCTT	204
**Cytochrome *c* oxidase complex biogenesis operon**				
AFE_3141	*ctaR*	TATGCCCTGCGGAGTCTAAT	TATTTAGGGACTCGGCACCA	202
**Sensor/regulator two component signal transduction system**				
AFE_3137	*regA*	CGATTGAGGCCATAAAGCTC	CAGCCGATATGTTGCCATTA	195
***petI* operon**				
AFE_3108	*sdrA1*	GTTTGGGTGCTGAAGTAGTG	GCAACAGTGGCAAACAGGC	318
AFE_3109	*petA1*	CCATTGAACCAGGTATGCAG	ATCCCGACATACCATTCAGG	202
AFE_3110	*petB1*	TTCCTTATTATTCTCGCAGCC	AAACCCCCGCAACAATAAGC	472
AFE_3111	*petC1*	GCCTCCTCCAAACTTGAGTC	TTCCGGGAAAAATTACTTGC	197
**Sulfide-quinone reductase**				
AFE_1792	*sqr*	AGACCGGTTATATGATCGAA	ATCATCTTGAACAGCACCTT	298
**Thiosulfate-quinone oxidoreductase complex operon**				
AFE_0043	*(doxDAII)*	ATAGACAATCCGCAACTCAC	ATGATGGTCAGATCATTGGT	302
AFE_0045	*(doxDAII)*	ATCAGAAACCCTTTCCCTAC	GTCGGACACATGAGGATAAC	298
AFE_0049	*(doxDAI)*	CAAAGGTCTGGATTTTACCA	ACCGTGCTGAACGTAATAGT	310
**Cytochrome *bd* ubiquinol oxidase**				
AFE_0955	*cydA*	AAGTTTGTGCATACCTCCAT	AGATTCTTCTGTTCGCTACG	307
**Cytochrome *bo* $_3$ ubiquinol oxidase**				
AFE_0632	*cyoB*	CAATTACATGGTCCACAACA	AGCGTATACACCACGATACC	300
**Heterodisulfide reductase complex operon**				
AFE_2553	*hdrA*	AGGAAATTTCTCCGAGTACC	GTGTTTTTCCAGACCGATAC	299
AFE_2555	*hdrC*	GAGATTGTGCAGTTGTTGTG	CTTCCACCTTCTGGATATTG	307
AFE_2586	*hdrB*	GGAGAAGGTCAAGAAGATCC	TACCAGGTGGAGTAGTCGAT	303
**Others**				
AFE_0282	*fur*	TACGAGCGAATGATCGACG	CACCATATGATCGTGGTGG	294
AFE_0445	*galU*	ATGGCTGAAGTGCGCAAGGC	ATGGTACTGACGTGGCTGGG	302
AFE_0539	*cysN3*	TCCGTGCTACAGGATATGC	GATATTCGGGCGGAAATTCG	337
AFE_1342	*epsS*	GCAACAACGTGGCGAACAC	AGGGGATCAGATGGCTCTC	306
AFE_1663	*glcF*	TTCCATCTGCTGGTACGAC	AGTTGTGCGCTTCGTTGTG	295
AFE_1677	*cbbOIa*	ACGGCATTGAGCTATACCG	TCCGGGTCTAGTAGTGCAT	265

AFE_2157	*cbbOII*	TATCTGAACACGCTACGCG	ACAGGTCGAGTTTGCGTTC	295
AFE_2324	*pgm*	GATCATGCACAGCATTGCCGC	TCAGGTGATGACAGTGGGG	352
AFE_2599	-	GAGATTGTCCAGTCACAGAG	CCTGCTCAAAGATCTGGTAG	262
AFE_2602	-	AGATCAGGGAGCTACAAGCG	TATCCTGACCGATTTCCTCCG	245
AFE_2836	*glbB*	CATTTCCTCAGTTACGCGG	TATAAGTGCCCCAGTCGCT	340
AFE_2840	*malQ*	CGAAGATCTGCGTTATCGC	TCGTTCATCTCGCTGTAGG	297
AFE_2971	*cysN2*	CTACGGTCCAGCAGATATTC	GCGATATCGTTCATCTGTAGC	322
AFE_3054	*cbbOIb*	CAGCGGTGACTTTGAAGATC	ATGAAAAGGCAGCCAGATGG	286
AFE_3116	-	CCGCCATATATTTGGCAGAG	CCTGCTCAAAGATCTGGTAG	262
AFE_3119	-	CGGAAAACATCCGAGCCTC	TATCCTGACCGATTTCCTCCG	198
AFE_3124	*cysD*	CTAGCAATCACCATCTGGAC	CATTACGCGAATCGCTTGC	327
